# A 24‐h helpline for patients undergoing oral food immunotherapy: Lessons from Italian experience during the SARS‐CoV‐2 pandemic

**DOI:** 10.1002/clt2.12251

**Published:** 2023-06-12

**Authors:** Laura Polloni, Antonella Muraro, Audrey DunnGalvin, Roberta Bonaguro, Francesca Lazzarotto, Laura Morandini, Rossana Schiavo, Alice Toniolo

**Affiliations:** ^1^ Food Allergy Referral Centre Veneto Region Department of Women and Children Health Padua University Hospital Padua Italy; ^2^ Psychology Unit Padua University Hospital Padua Italy; ^3^ Department of Paediatrics and Child Health School of Applied Psychology University College Cork Cork Ireland

To the Editor,

The COVID‐19 outbreak has substantially impacted people's lives, especially for more vulnerable individuals.^1^, ^2^ Among patients with food allergy (FA), difficulties accessing “safe” foods and health services have been commonly reported, with significantly overall poorer subjective wellbeing.^2^ Patients with FA undergoing oral immunotherapy (OIT) may have been particularly affected by the pandemic since the protocol requires many in‐hospital visits to gradually increase the allergen ingestion due to the risk of severe reactions.^3^ Such a risk can be a source of anxiety for patients and families.^4^, ^5^

The COVID‐19 pandemic led to an unprecedented change in clinical procedures, with fast implementation of telemedicine programs.^6^ Telemedicine has been reported as a beneficial means to support allergic patients, including those undergoing OIT.^1^ Italy was the first European country impacted by the pandemic and Veneto was the region hit fourth hardest. Since 2011, in our Food Allergy Centre in Padua, an expert support telephone line is accessible 24/7, for patients undergoing OIT. This cross‐sectional retrospective survey aimed to evaluate patients/parents attitudes and satisfaction about the service during the first year of the pandemic in the Veneto region. We wanted to better understand participants' needs and perceived benefits concerning the helpline to continue to support patients post pandemic with more tailored and effective interventions.

In March 2021, adults and parents of a child medically diagnosed with IgE‐mediated FA undergoing OIT were asked to complete an online survey on their experiences of using and satisfaction with the helpline over the last 12 months. From a sample of 110 eligible participants, 85 (77%) accepted giving written consent. The Padua University Hospital Ethics Committee approved the study. The survey evaluated user experience, specifically (a) Reasons for using the helpline, (b) Alternative actions if the service was not available and (c) Satisfaction with the service. Reliability for “Satisfaction” was found excellent (*α* = 0.94). Since Spearman correlations between the items ranged from *r* = 0.82–0.97, we created a composite mean Satisfaction score. We performed the chi–square test for categorical variables and *t*‐tests or ANOVA for continuous dependent variables. A simple logistic regression examined the effect of the OIT phase and a linear regression model factors predicted helpline use.

Sixty‐five parents and 20 adult patients completed the survey (Table 1), 83% female. Patients had mean age 15 years, (SD = 8.22), 57% male. OIT was for milk (*N* = 58, 68.2%), egg (*N* = 23, 27.1%), and wheat (*N* = 4, 4.7%); up‐dosing phase (*N* = 56, 68%), and maintenance (*N* = 29, 34.1%). Fifty‐four percent called the helpline at least once. There was no significant relationship (*p* < 0.05) between sex and helpline access. No significant differences were found between the user/non‐user groups on education, age, FA number, or OIT allergen. A significant difference was found for the user groups OIT phase (*t* = 2.69; *p* = 0.009). More calls were made in the up‐dosing (*N* = 36, 78%, *M* = 1.2; SD = 1.0) compared to the maintenance phase (*N* = 10, 22%, *M* = 0.69; SD = 1.0). In logistic regression, those in up‐dosing were over three times as likely to call the helpline as those in maintenance (OR = 3.6, *p* = 0.04; 95% CI 1.1–12.2). Concerning the reasons why the helpline was accessed and alternative actions if the service had not been available (Table 2), “clarifications” was the most frequent reason cited (70%), followed by “mild/moderate symptoms” (57%); 35% “reassurance” and 11% “suspected anaphylaxis.” If not available, the majority (87%) would have visited their physician (49%) or the hospital (38%).

**TABLE 1:** Profile of respondents and patients undergoing OIT, split by those who called and those who did not call the helpline.

Parent^a^				Patient^a^			
	Gender^b^	Age^b^	Education^b^	Gender	Age	OIT food	OIT phase
Did not call	Male, *n* = 6, 15.4%	Mean age of 47.40 (SD = 5.51)	Middle school, *n* = 3, 7.7%	Male, *n* = 25, 64.1%	Mean age of 15.36 (SD = 9.41)	Milk, *n* = 25, 64.1%	Up‐dosing, *n* = 20, 51.3%
	Female, *n* = 24, 61.5%		High school, *n* = 12, 30.8%	Female, *n* = 14, 35.9%		Egg, *n* = 13, 33.3%	Maintenance, *n* = 19, 48.7%
	Total, *n* = 30, 76.9%		University, *n* = 16, 41.0%	Total, *n* = 39, 100%		Wheat, *n* = 1, 2.6%	Total, *n* = 39, 100%
	Missing, *n* = 9, 23.1%		Total, *n* = 31, 79.5%			Total, *n* = 39, 100.0%	
			Missing, *n* = 8, 20.5%				

	Gender^b^	Age^b^	Education^b^	Gender	Age	OIT type	OIT phase
At least once	Male, *n* = 5, 10.9%	Mean age of 45.22 (SD = 6.95)	Middle school, *n* = 1, 2.2%	Male, *n* = 23, 50%	Mean age of 14.50 (SD = 7.14)	Milk, *n* = 33, 71.7%	Up‐dosing, *n* = 36, 78.3%
	Female, *n* = 30, 65.2%		High school, *n* = 20, 43.5%	Female, *n* = 23, 50%		Egg, *n* = 10, 21.7%	Maintenance, *n* = 10, 21.7%
	Total, *n* = 35, 76.1%		University, *n* = 16, 34.8%	Total, *n* = 46, 100%		Wheat, *n* = 3, 6.5%	Total, *n* = 46, 100%
	Missing, *n* = 11, 23.9%		Total, *n* = 37, 80.4%			Total, *n* = 46, 100.0%	
			Missing *n* = 9, 19.6%				

^a^ Mean or *N* and percentage, as appropriate.

^b^ A number of participants (*n* = 19 in total) chose not to provide age, gender and/or education.

**TABLE 2:** Participants' responses to the survey: (a) Reasons for accessing helpline and (b) actions if helpline was not available; (c) satisfaction with the 24 h helpline on six items and Satisfaction Global Score.

(a) Reason(s) for accessing helpline^a^	*N*	%
Mild/moderate allergic symptoms	26	56.5
Ongoing anaphylaxis	5	11.1
Reassurance	16	34.8
Clarifications	32	69.6
Other	0	0
(b) Action(s) if helpline was not available^a^	*N*	%
Waited for your next appointment	4	8.9
Visited your physician	22	48.9
Visited the hospital	17	37.8
Done nothing	1	2.2
Taken other action	12	26.1
(c) Satisfaction^b^	Mean (SD)	*R* ^c^
1. Were your questions answered well?	4.93 (0.33)	0.97
2. Was the advice given helpful?	4.93 (0.33)	0.97
3. Did you follow the advice?	4.96 (0.21)	0.96
4. Did you feel more confident in dealing with the issue about which you rang?	4.91 (0.29)	0.82
5. Would you ring the helpline again?	5.00 (0.01)	0.97
6. Overall, how valuable to you is the helpline service for OIT patients?	5.00 (0.01)	0.98
Satisfaction Global Score	4.96 (0.18)	

^a^ Respondents could choose more than one option so the total does not necessarily add to 100%.

^b^ Response scale from 1 = not at all to 5 = extremely.

^c^ *p* < 0.001 for all items.

Users reported a very high level of satisfaction (*M* = 4.96; SD = 0.18). A strong correlation was found between items and composite Satisfaction (*p* < 0.001).

The overall regression model was significant (*F* = 5.27; *p* < 0.001) with the number of calls predicting satisfaction (*β* = 0.14, *p* ≤ 0.006; 95% CI 0.1–0.23).

OIT is a challenging process for patients and their families. It is prolonged and requires daily allergen consumption, and adverse reactions are often experienced.^4^, ^7^ The 24/7 availability of experts can provide important support that makes a difference for patients/families. In the present study, more than half of the respondents used the service at least once, with very high satisfaction. The more often respondents called, the more satisfied they were. Most users called the service to clarify issues and concerns around OIT. Telehealth methods may present a resource‐friendly complementary way to ensure that families are safely monitored, provided with continuing support and education, and that parental/patient concerns are addressed. Support is essential for maintaining safety and long‐term success.^8^ Although additional research is needed, the helpline may facilitate adherence by quickly addressing concerns around side effects, and related issues. Indeed, patients in the up‐dosing phase were over three times as likely to call as those in the maintenance phase. This result may suggest when support is needed most, helping to prevent drop‐out or dissatisfaction with treatment. These findings are also in line with a previous study reporting that 24‐h telephone access to experts significantly improved the disease‐specific quality of life of children and families with life‐threatening FA compared with usual clinical care.^9^

We acknowledge some limitations of this study: firstly, an ad‐hoc measure was used, although we found excellent Cronbach's alpha. Secondly, the sample size was relatively small and self‐selected; however, the participation of 77% may reduce potential bias.

Evaluating new ways to promote the continuity and safety of care is crucial. A 24/7 expert support telephone line for patients undergoing OIT has proved to be useful to support patient and family adherence and compliance, while also minimizing hospital access. Therefore, these services should be integrated more widely on a permanent basis for FA care, including OIT.^6^ The present findings can increase understanding or ways to potentially enhance OIT care pathways in the post‐pandemic era.

## AUTHOR CONTRIBUTIONS

**Laura Polloni**: Conceptualization (equal); data curation (equal); formal analysis (equal); investigation (equal); methodology (equal); project administration (lead); supervision (equal); writing – original draft (lead); writing – review & editing (equal). **Antonella Muraro**: Conceptualization (equal); investigation (equal); methodology (equal); project administration (lead); resources (lead); supervision (lead); writing – original draft (equal); writing – review & editing (equal). **Audrey DunnGalvin**: Conceptualization (equal); data curation (equal); formal analysis (lead); investigation (equal); methodology (equal); supervision (equal); writing – original draft (equal); writing – review & editing (equal). **Roberta Bonaguro**: Data curation (equal); writing – review & editing (equal. **Francesca Lazzarotto**: Data curation (equal); writing – review & editing (equal). **Laura Morandini**: Data curation (equal); writing – review & editing (equal). **Rossana Schiavo**: Supervision (equal); writing – review & editing (equal). **Alice Toniolo**: Data curation (equal); writing – review & editing (equal).

## CONFLICT OF INTEREST STATEMENT

Nothing to declare.
